# Crop coefficient determination and evapotranspiration estimation of watermelon under water deficit in a cold and arid environment

**DOI:** 10.3389/fpls.2023.1153835

**Published:** 2023-06-16

**Authors:** Hengjia Zhang, Zeyi Wang, Shouchao Yu, Anguo Teng, Changlong Zhang, Lian Lei, Yuchun Ba, Xietian Chen

**Affiliations:** ^1^ College of Agronomy and Agricultural Engineering, Liaocheng University, Liaocheng, China; ^2^ Yimin Irrigation Experimental Station, Hongshui River Management Office, Zhangye, China; ^3^ College of Water Conservancy and Hydropower Engineering, Gansu Agricultural University, Lanzhou, China

**Keywords:** water stress, reference crop evapotranspiration, water consumption characteristics, meteorogical factors, watermelon, leaf area index

## Abstract

To investigate the evapotranspiration and crop coefficient of oasis watermelon under water deficit (WD), mild (60%–70% field capacity, FC)and moderate (50%–60% FC) WD levels were set up at the various growth stages of watermelon, including seedling stage (SS), vine stage (VS), flowering and fruiting stage (FS), expansion stage (ES), and maturity stage (MS), with adequate water supply (70%–80% FC) during the growing season as a control. A two-year (2020-2021) field trial was carried out in the Hexi oasis area of China to explore the effect of WD on watermelon evapotranspiration characteristics and crop coefficient under sub-membrane drip irrigation. The results indicated that the daily reference crop evapotranspiration showed a sawtooth fluctuation which was extremely significantly and positively correlated with temperature, sunshine hours, and wind speed. The water consumption during the entire growing season of watermelon varied from 281–323 mm (2020) and 290–334 mm (2021), among which the phasic evapotranspiration valued the maximum during ES, accounting for 37.85% (2020) and 38.94% (2021) in total, followed in the order of VS, SS, MS, and FS. The evapotranspiration intensity of watermelon increased rapidly from SS to VS, reaching the maximum with 5.82 mm·d^-1^ at ES, after which it gradually decreased. The crop coefficient at SS, VS, FS, ES, and MS varied from 0.400 to 0.477, from 0.550 to 0.771, from 0.824 to 1.168, from 0.910 to 1.247, and from 0.541 to 0.803, respectively. Any period of WD reduced the crop coefficient and evapotranspiration intensity of watermelon at that stage. And then the relationship between *LAI* and crop coefficient can be characterized better by an exponential regression, thereby establishing a model for estimating the evapotranspiration of watermelon with a Nash efficiency coefficient of 0.9 or more. Hence, the water demand characteristics of oasis watermelon differ significantly during different growth stages, and reasonable irrigation and water control management measures need to be conducted in conjunction with the water requirements features of each growth stage. Also, this work aims to provide a theoretical basis for the irrigation management of watermelon under sub-membrane drip irrigation in desert oases of cold and arid environments.

## 1 Introduction

Desert oases are an essential barrier to guaranteeing regional food and ecological security. In the interior of northwest China, the oasis agricultural region of Hexi features a temperate continental desert climate with little to no annual rainfall and a severe water resource constraint (Deng et al., 2017). As is well known, oasis agriculture is dominated by irrigated agriculture, so without irrigation, there would be no agriculture (Li et al., 2016). The proportion of agricultural water use in the total water resources of the Hexi area is the largest, at 88.40% (Wang et al., 2019). At present, agricultural cultivation in most areas of the Hexi Oasis region still uses the traditional check-field flood irrigation, with its backward and unreasonable irrigation techniques and low water use efficiency (WUE) (Wang et al., 2020a). Moreover, together with the long years of machine well extraction, this has led to the overexploitation of local groundwater resources, aggravating the degree of arable desert and deteriorating the ecological environment (Chai et al., 2014). Although, the area has long sunshine hours, a short frost-free period, abundant light and heat resources, and a 15–20°C temperature difference between day and night, all of which make it an ideal base for melon cultivation (Yang et al., 2018; Li et al., 2022a). A shortage of water resources and water waste in the region (Huang et al., 2017; Zhou et al., 2022), along with the recent blind expansion of watermelon and other specialty cash crops, has severely hampered the development of the local watermelon industry. Thus, studies on water consumption and evapotranspiration of oasis watermelon under water deficit conditions are of vital practical value for water resources deployment and to ensure stable and high watermelon yields.

The production of special melons in the oasis is a highly advantageous enterprise. But the watermelon’s growth can be significantly affected by changes in water availability because of its high water requirements and sensitivity to soil moisture (Leskovar et al., 2007; Zhang et al., 2009). During certain growth periods maintaining an appropriate water deficit (WD) not only enhances the conversion of photosynthetic products to fruits but also suppresses excessive nutrient growth in the above-ground parts and promotes root growth, thereby improving yield and quality (Rahmati et al., 2018; Zuniga et al., 2018). The results of a study using daily evaporation from the water surface of the evaporation dish as the water control criterion showed that the overall benefits for watermelon were best when the evaporation coefficients were determined at 0.75, 0.75, 1.25, and 1.00 for the seedling (SS), flowering and fruiting (FS), expansion (ES) and maturity (MS) stages in that order (Lin et al., 2010). As for the study that used drip irrigation frequency as a water control criterion, the recommended drip irrigation system for greenhouse watermelon in the arid zone was one drip every four days at both SS and ES, while one drip every two days at FS and one drip every six days at MS (Liu et al., 2014). In addition, the results of the experiment by Wu and Wang (2008) showed that soil water content (SWC) in watermelon fields was controlled at 70.92%–81.24% while leaf area index (*LAI*) was between 1.44–1.32, which both enhanced leaf photosynthetic efficiency and promoted the accumulation of assimilates. Kuscu et al. (2015) achieved an optimum balance between water productivity, quantity, and quality in watermelon fruits under semi-humid conditions with 100% crop evapotranspiration (*ET_c_*) before maturity and 50% *ET_c_* recovery afterward. Accordingly, by scientifically managing the water demand pattern during the watermelon’s growing season in the oasis, high-quality and efficient ecological watermelon cultivation may be achieved.

Evapotranspiration (*ET*) is a critical component of farmland water balance during the hydrologic cycle, which has a significant effect on crop development and yield (Cui et al., 2008b). The water in farmland under sub-membrane drip irrigation is mainly consumed by *ET_c_* (Skaggs et al., 2010; Feng et al., 2018). Thus, accurate estimation of *ET_c_* is crucial to reducing water consumption during the crop reproductive period, improving WUE, and developing water-saving agriculture (Liu et al., 2002; He et al., 2013). There are two main methods for calculating *ET_c_*: one is a direct calculation approach, and the other uses the reference crop evapotranspiration (*ET*_0_) and the crop coefficient (*K_c_*). Since most of the direct calculation methods are empirical formulas with strong geographical limitations, the prevailing method is based on *ET*_0_ (Liu et al., 2009). Related studies have indicated that the *ET*_0_ calculated using the Penman-Monteith (P-M) is the closest to the measured values in both humid and arid regions, so it is a preferred calculation method that is widely used (Allen et al., 1989; Allen et al., 2011).

The *LAI* is an essential growth indicator that reflects the quality of crop population and is strongly linked to photosynthesis, transpiration, and water productivity (Zhao et al., 2018; Wang et al., 2021). A study has shown that variation in plant leaf area has a substantially greater effect on evapotranspiration than that of variation in the climate and that climatic factors have a stronger influence on evapotranspiration indirectly through plant cover (Yang et al., 2022). As well known, *K_c_* is relevant to *LAI* dynamics, intercepted solar radiation, and crop phenological stage (Netzer et al., 2009). Meanwhile, crop stage water requirement is equal to the product of stage *ET*_0_ and *K_c_* (Meng, 2011).

For apple trees under irrigated conditions, Du et al. (2017) discovered a positive correlation between transpiration and *LAI*, while Juhasz and Hrotko (2014) reached the same conclusion for the Hungarian sweet cherry. Munitz et al. (2019) reported a significant linear relationship between *LAI* and *K_c_* for irrigated vineyards, i.e., *K_c_* could be estimated by measuring the *LAI* of the vineyard and combining it with meteorological data from neighboring weather stations to calculate *ET*_0_, and then *ET_c_* is obtained. As can be seen, it is feasible to establish a regression model of *LAI* against *K_c_* (Zhang et al., 2019). However, *K_c_* is greatly influenced by environmental conditions such as climate, soil, and degree of WD in different areas (Wang et al., 2020b). Therefore, investigating the relationship between *LAI* and *K_c_* under WD conditions is critically valuable for accurately predicting oasis watermelon *ET* in the Hexi region.

In conclusion, a model can be established to estimate the *ET* of oasis watermelon in the Hexi region using *LAI* and meteorological factors, thereby letting the model to master the dynamic water consumption law of local watermelon during various growth periods, which has excellent practical significance for enhancing the level of watermelon precision management, formulating a reasonable water dynamic management plan, and maintaining the stability of fruit yield. However, there exists a seriously limited knowledge in the Hexi region about the water consumption characteristics of watermelon under mulched drip irrigation, and studies on the determination of *K_c_* and *ET* estimation models for oasis watermelon have not been reported. Therefore, the aims of this study were: (1) to analyze the stages evapotranspiration characteristics of oasis watermelon under WD and its change law; (2) to accurately calculate *ET*_0_ during the reproductive period of watermelon based on the PM equation and then invert *K_c_* of oasis watermelon; and (3) to establish an estimation model of oasis watermelon evapotranspiration, thereby achieving precise irrigation at various growth phases of oasis watermelon and effectively improving the water productivity in the Hexi region.

## 2 Materials and methods

### 2.1 Description of the study site

The experiment was conducted from May to August 2020 and 2021 at the Yimin Irrigation Experiment Station (100°43′ E, 38°39′ N, altitude: 1,970 m, total area: 20 mu), which is a joint scientific research and training base of Gansu Agricultural University. The test site is in a typical desert oasis of China’s northwestern arid region, where the annual average temperature is 6°C, the frost-free period is 165 days, the annual precipitation mostly ranges between 183–285 mm, the annual average evaporation is 2,000 mm, the annual average sunshine hours is 3,000 h, and the climate is temperate continental grassland. The soil texture of the test site is a light loam with a soil bulk density of 1.4 g·cm^-3^ and a pH of 7.2. The field capacity (FC) of tilled soil is 24.0% (mass water content), and the wilting point is 8.2%. The salinization hazard is negligible due to the large depth of groundwater burial.

### 2.2 Experimental design and agronomic management

The watermelon variety tested was the “Xinong 8,” a local main planting. The main growth period of oasis watermelon was divided into five stages: seedling stage (SS), vine stage (VS), flowering and fruiting stage (FS), expansion stage (ES), and maturity stage (MS). Deep plowing, weeding, fertilizer application, drip irrigation, and mulching were carried out before sowing so that the initial conditions of water and nutrients in each plot were similar. Planting was done manually by breaking the film in a north-south direction, with two rows at 30 cm × 210 cm spacing in each plot. The irrigation method was sub-membrane drip irrigation, with one pipe controlling one row of crops and the length of the pipe equaling the length of the plot. Specific agronomic management measures are presented in **Table 1**.

**Table 1:** Agronomic management practices in the oasis watermelon trial plots.

Agronomic practices	2020	2021
Basal fertilizer	Ternary compound fertilizer (N+P_2_O_5_+K_2_O, 15-15-15) 760 kg·hm^-2^ (Shandong Xianglong Chemical Fertilizer Co., Ltd.) applied in open furrows according to local standards	
Drip irrigation	Inner patch type drip irrigation pipe (tube diameter, wall thickness, and drip hole spacing are de16(inner), 0.2, 300, mm) (produced by Gansu Haina Plastic Industry Co.)	
Film	Polyethylene blow molded agricultural colorless mulch, width 750 mm, thickness 0.01 mm (Lanzhou Fluor Plastics Co., Ltd.)	
Planting mode	Flat mulching, north-south orientation	
Sowing dates	30 April	1 May
Sowing method	Manually spotted and covered with a little sand	
Sowing depth	2–3 cm	2–3 cm
Plant spacing	30–35 cm	30–35 cm
Row spacing	210 cm	210 cm
Harvesting dates	19 August	15 August
Field management	Weeding, grooming, and topping were all conducted promptly, with consistency in the manner and timing of each treatment.	

The experiment was a one-way completely randomized design. WD treatments were applied mainly at the watermelon SS, VS, ES, and MS. The amount of irrigation was calculated as the average SWC as a percentage of the FC in the planned wet layer (0–60 cm), and irrigation was applied immediately to the upper limit when the measured SWC was below (or close to) the design lower limit of the treatment. A total of nine treatments were set up, with three replications of each treatment, in which suitable irrigation was performed throughout the reproductive period and served as the control treatment. The specific experimental design scheme is illustrated in **Table 2**.

**Table 2:** The experimental design scheme of oasis watermelon.

Treatments	Processing name	Water deficit treatments at different growth stages (percentage of field capacity)				
		SS	VS	FS	ES	MS
CK	Control treatment	70–80	70–80	70–80	70–80	70–80
WD1	Mild at the seedling stage	60–70	70–80	70–80	70–80	70–80
WD2	Moderate at the seedling stage	50–60	70–80	70–80	70–80	70–80
WD3	Mild at the vine stage	70–80	60–70	70–80	70–80	70–80
WD4	Moderate at the vine stage	70–80	50–60	70–80	70–80	70–80
WD5	Mild at the expansion stage	70–80	70–80	70–80	60–70	70–80
WD6	Moderate at the expansion stage	70–80	70–80	70–80	50–60	70–80
WD7	Mild at the maturity stage	70–80	70–80	70–80	70–80	60–70
WD8	Moderate at the maturity stage	70–80	70–80	70–80	70–80	50–60

Numerical values are the percentage of field capacity. SS, seedling stage; VS, vine stage; FS, flowering and fruiting stage; ES, expansion stage; MS, maturity stage.

### 2.3 Measurements and calculations

#### 2.3.1 Meteorological information

Meteorological data was achieved from a fully automatic weather station located at the experimental site. Meteorological factors such as precipitation (*P*), air temperature (*T*), relative humidity (*RH*), sunshine hours (*n*), wind speed (*u*), wind direction, and air pressure were automatically measured in the test area.

#### 2.3.2 Soil water content (SWC)

The SWC of the planned wet layer (sampled every 20 cm) was determined layer by layer using the dry method every 7–10 days after the start of the treatment, with the sampling points randomly selected in the middle of two watermelon plants and the average value taken to determine the amount and timing of irrigation, with additional measurements before and after irrigation.

#### 2.3.3 Leaf area index (*LAI*)

The non-linear regression equation for single leaf area (*LA*) against leaf width (*w*) was established according to the reference (Zheng, 2009): *LA* = exp^-0.096^ *w* ^1.758^, where the determination coefficient R^2^ = 0.912 (*p*< 0.01). The *LAI* was gained by calculating the total *LA* of all plants per unit of land and then comparing it to the land area.

#### 2.3.4 Crop evapotranspiration

Crop evapotranspiration (*ET_c_*) was calculated by using the water balance equation (Cui et al., 2008a).


(1) $${\text{ET}}_{\text{c}}{\text{ = P}}_{\text{r}}\text{ + U + I }-\text{D }-\text{S }+\Delta \text{W}$$

where *P_r_* is the effective precipitation in each period (mm); *U* is the groundwater recharge (mm), the test site is in an inland arid area, the groundwater burial depth is below 20 m, so the groundwater recharge can be neglected, i.e., *U*=0. *I* is the amount of irrigation water during the period (mm); *D* is the amount of deep seepage (mm), because the soil moisture design is lower than the field capacity, and the design flow rate of under-film drip irrigation is 2 L·h^-1^, there is no deep seepage problem, so the amount of deep seepage is negligible, i.e., *D*=0; *S* is the surface runoff (mm), the test field is in flat mulching, there is no surface runoff, i.e., *S*=0; *ΔW* is the change of soil water storage in the 0–100 cm-layer during the period (mm).

Hence, Eq. (1) can be simplified as follows.


(2) $${\text{ET}}_{\text{c}}{\text{ = P}}_{\text{r}}\text{ + I }+\;\Delta \text{W}$$

*ΔW* and I in Eq. (2) are calculated as follows.


(3) $$\Delta W=10\rho_{i}\sum^{}\left(\theta_{i1}-\theta_{i2}\right)H_{i}$$

(4) $$I=10\rho Hp\left(\theta_{max}-\theta_{t}\right)/\eta$$

where *θ_i_*_2_ and *θ_i_*_1_ are the volumetric SWC of *i*-layer at the end and beginning of the calculation period, respectively (%); *H_i_* is the thickness of *i*-layer (mm); *I* is the volume of single irrigation (mm); *ρ* is the soil bulk density (g·cm^-3^); *H* is the depth of the plan wetted layer (cm); *p* is the design wetting ratio, 0.65 is taken for this test; *θ_max_* is the design upper limit of SWC (%); *θ_t_* is the SWC before irrigation (%); *η* is the irrigation water utilization ratio, taken as 0.95 in this experiment.

The stage evapotranspiration intensity and water consumption modulus can be found in Wei et al. (2017).

#### 2.3.5 Reference crop evapotranspiration (*ET*_0_)

*ET*_0_ was calculated using the Penman-Monteith (P-M) formula, strongly recommended by FAO 1998 (Allen et al., 1998).


(5) $$ET_{0}=\frac{0.408\Delta \left(R_{n}-G\right)+\gamma \frac{900}{T+273}u_{2}\left(e_{s}-e_{a}\right)}{\Delta +\gamma \left(1+0.34u_{2}\right)}$$

where *ET*_0_ is the reference crop evapotranspiration (mm); *R_n_* is the net radiation (MJ·m^-2^·d^-1^); *G* is the soil heat flux (MJ·m^-2^·d^-1^); *Δ* is the slope of the saturation water vapor pressure against temperature curve (kPa·°C^-1^); *γ* is the hygrometer constant (kPa·°C^-1^); *T* is the mean air temperature (°C); *u*_2_ is the wind speed at a height of 2 m above ground (m·s^-1^); *e_s_* is the saturation water vapor pressure of air (kPa); *e_a_* is the actual water vapor pressure of air (kPa).

#### 2.3.6 Crop coefficient

The crop coefficient (*K_c_*) under no WD conditions is determined by the ratio of evapotranspiration under adequate water supply conditions to the ET_0_. This is calculated by Eq. (6).


(6) $$K_{c0}=ET_{c}/ET_{0}$$

where *K_c_*_0_ is the *K_c_* without water stress; *ET_c_* is the evapotranspiration under adequate water supply (mm); *ET*_0_ is the reference crop evapotranspiration (mm).

When soil moisture is insufficient, the *K_c_* is not only affected by meteorological factors and the crop’s characteristics but also by the SWC. Therefore, the moisture stress coefficient is introduced into the calculation of the *K_c_*. The moisture stress coefficient is derived from the evapotranspiration measured under WD conditions, the *ET*_0_, and the *K_c_*_0_ (Allen et al., 1998) and is calculated as follows.


(7) $$K_{s}=ET_{WD}/\left(ET_{0}\times K_{c0}\right)$$

where *K_s_* is the water stress coefficient; *ET_WD_* is the evapotranspiration under water stress conditions (mm); *ET*_0_ is the reference crop evapotranspiration (mm).

The *K_c_* is calculated as follows.


(8) $$K_{c}=K_{c0}\times K_{s}$$

#### 2.3.7 Methodology for testing of *ET_c_* estimation model

The correlation coefficient (*r*), Nash efficiency coefficient (NSE), and total equilibrium coefficient (*R*) were considered as statistical coefficients to evaluate the merits of the *ET* estimation model (Wang et al., 2014). The error analysis was performed quantitatively using average relative error (ARE) and mean absolute percentage errors (MAPE).

### 2.4 Data processing

The average and standard deviation (± SD) of the data were calculated in Microsoft Excel 2010 (Microsoft Corp., Raymond, Washington, USA). The software Origin 2020 (Origin Lab, Corp., Hampton, Massachusetts, USA) was used for graphing and regression analysis. Significance analysis with Duncan’s multiple range test was performed at a 5% level using SPSS 19.0 (IBM, Inc., New York, USA).

## 3 Results

### 3.1 Distribution characteristics of *ET*_0_

**Figure 1:**
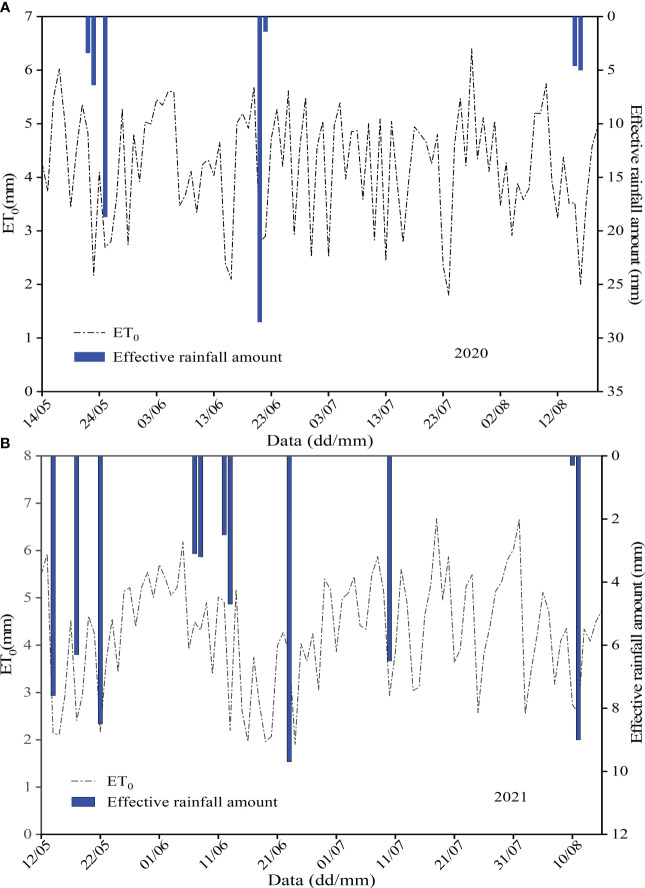
Variation of daily *ET*_0_ and effective precipitation distribution during the growing season of oasis watermelon in 2020 **(A)** and 2021 **(B)**.

The daily *ET*_0_ variation and effective rainfall distribution for oasis watermelon during two consecutive growing seasons from 2020–2021 are indicated in **Figures 1A, B**. We can see from **Figure 1**, the daily *ET*_0_ fluctuated in a sawtooth pattern with sharp fluctuations, especially in late July and early August, but the overall trend was relatively stable, with the peak occurring from late July to early August. Moreover, the higher *ET*_0_ was concentrated after the fruit set, among which the frequency of daily variation was higher in 2020 and reached a peak of 6.40 mm on 28 July, while the frequency of daily variation was relatively low in 2021 and reached a maximum of 6.66 mm on 1 August. This is mainly related to the climatic characteristics (temperature) of the trial site. Besides, the distribution of rainfall also showed that the greater the daily rainfall on a rainy day, the smaller the corresponding daily *ET*_0_, with a negative correlation between them.

As seen in **Figure 1**, the effective rainfall during the watermelon reproductive period in 2020 was 68 mm, and the rainfall during the SS, VS, FS, ES, and MS was 28.50 mm, 29.90 mm, 0.00 mm, 0.00 mm, and 9.60 mm, respectively, with the highest rainfall of 28.5 mm on 21 June. Similarly, it can be seen that the effective rainfall in 2021 for different growth stages and the whole reproductive period was 28.70 mm, 16.90 mm, 0.00 mm, 6.50 mm, 9.30 mm, and 61.40 mm, respectively. The largest amount of rainfall was 9.7 mm on 23 June. It can be concluded that irrigation should be controlled during the SS in watermelon cultivation to avoid excessive irrigation consuming the less water-demanding reproductive period, and on the contrary, timely irrigation is required during the melon ES to replenish soil water to avoid WD. It is also clear from **Figure 1** that the *ET*_0_ of stage cumulative was highest at the VS (128.32 mm in 2020 and 117.17 mm in 2021), while the *ET*_0_ of the FS was relatively lowest because of its shortest duration (19.30 mm in 2020 and 23.27 mm in 2021). The mean of daily *ET*_0_ results was highest at SS and lowest at FS in 2020, highest at ES and lowest at VS in 2021.

*ET*_0_ and its composition for different growing periods of oasis watermelon in the Hexi region are illustrated in **Table 3**. The *ET*_0_ calculated by the P-M equation is derived from the sum of the solar radiation term (*ET_rp_*) and the aerodynamic term (*ET_op_*), both of which constitute the effect on the *ET*_0_ (Glasbey and Allcroft, 2008). As can be seen in **Table 3**, the proportion of *ET_rp_* in each stage of oasis watermelon (in 2020, for example) was 71.48%, 76.67%, 80.09%, 78.14%, and 75.97%, in that order. The proportion of *ET_rp_* in *ET*_0_ showed a single-peak trend of increasing and then decreasing as the growth period progressed and increased to the largest proportion at FS and ES. Meanwhile, the weight of *ET_rp_* influencing *ET*_0_ was greater than 70%, much higher than *ET_op_*, while the total *ET_rp_* share was 75.60% throughout the growing season. This is mainly because the respective ratios of *ET_rp_* and *ET_op_* are affected by the regional geography and climatic environment and change over time. The Hexi Oasis lies in the interior, where there is an enormous amount of *ET_rp_* rather than *ET_op_* in *ET*_0_ due to the region’s arid environment, lack of precipitation, high evaporation intensity, and long daylight hours.

**Table 3:** Cumulative *ET*_0_ and its composition for oasis watermelon.

Year	Growth stage	*ET_rp_*/mm	*ET_op_*/mm	*ET_0_*/mm	Daily *ET*_0_ mean/mm
2020	SS	86.75	34.61	121.36	4.33
	VS	98.39	29.94	128.32	4.28
	FS	15.46	3.84	19.30	3.86
	ES	78.77	22.03	100.80	4.20
	MS	33.90	10.71	44.62	4.06
	The entire growing season	313.26	101.13	414.39	4.15
2021	SS	79.85	33.34	113.19	4.35
	VS	94.96	22.21	117.17	3.91
	FS	18.80	4.48	23.27	4.65
	ES	86.50	21.60	108.10	4.70
	MS	38.59	10.03	48.62	4.05
	The entire growing season	318.70	91.65	410.36	4.33

### 3.2 Effects of meteorological factors on *ET*_0_

The results of the *ET*_0_ calculation are directly influenced by the meteorological factors needed in the P-M equation. Due to the unique climatic conditions of the Hexi Oasis region, it is imperative to examine the correlations between the following meteorological variables: daily maximum temperature (*T_max_*), daily minimum temperature (*T_min_*), daily mean temperature (*T_mean_*), sunshine hours (*n*), atmospheric pressure (*P*), relative air humidity (*RH*), water vapor pressure (*e_a_*), wind speed (*u*_2_) and *ET*_0_, as well as the correlation between *ET*_0_ and meteorological data under the interaction of multiple factors. The meteorological data for 2021 was used as an example in the study that was done above.

**Figure 2:**
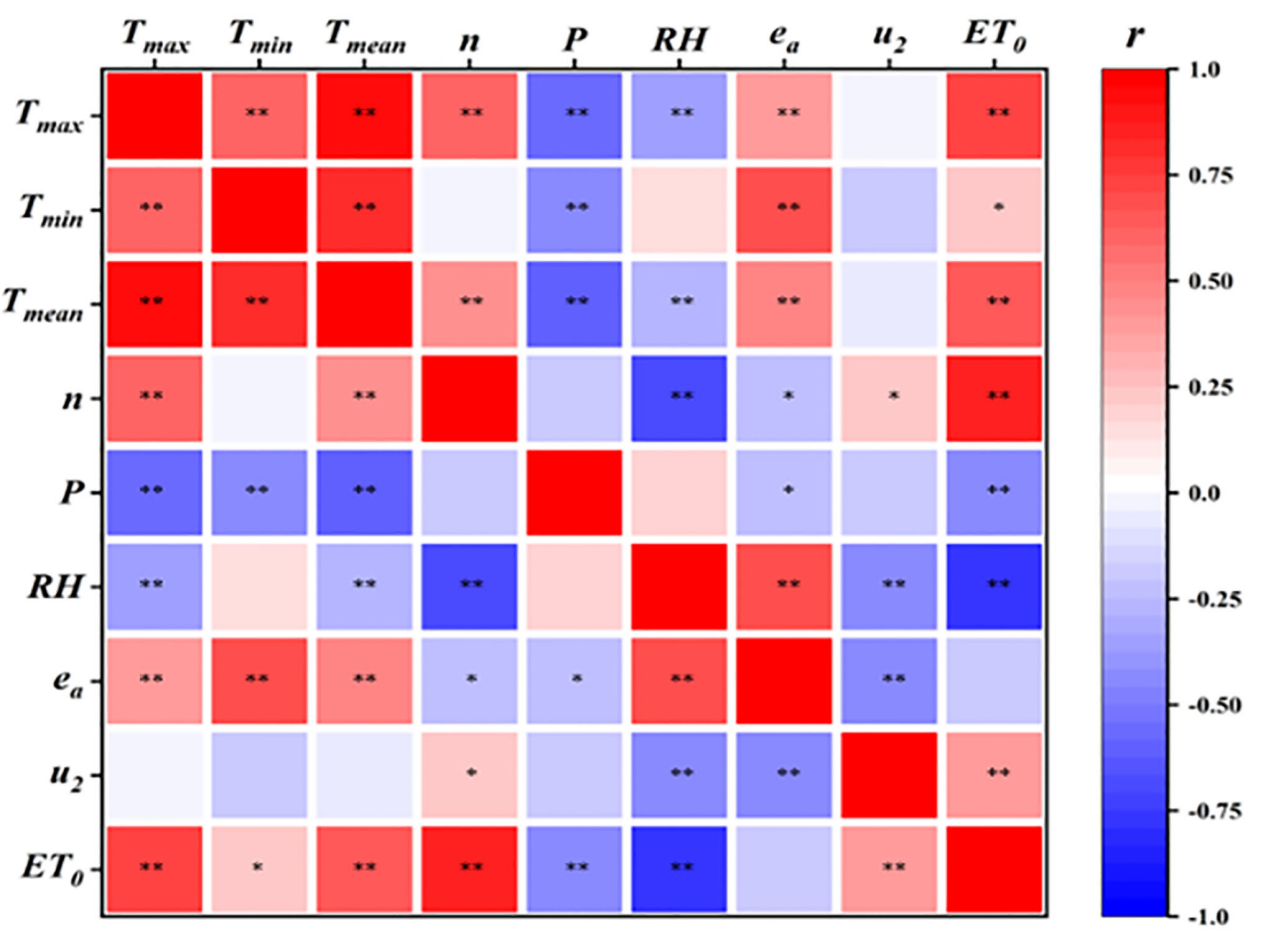
Correlation analysis of *ET*_0_ and meteorological elements during the watermelon growing period in 2021. Correlations between the two indicators were significant at *p< 0.05 and **p< 0.01. *T_max_*, daily maximum temperature; *T_min_*, daily minimum temperature; *T_mean_*, daily mean temperature; *n*, sunshine hours; *P*, atmospheric pressure; *RH*, relative air humidity; *e_a_*, water vapor pressure; *u*_2_, wind speed.

**Figure 2** presents the bivariate correlation analysis between meteorological factors and *ET*_0_ during the watermelon growing season. The Pearson correlation analysis shows that *ET*_0_ had a highly significant positive correlation with *T_max_*, *T_mean_*, *n*, and *u*_2_ whose coefficients were 0.721, 0.644, 0.870, and 0.392, respectively; a significant positive correlation with *T_min_* whose coefficient was 0.234; and a negative correlation with *P*, *RH*, and *e_a_* whose coefficients were -0.439, -0.761, and -0.195, which were negatively correlated.

**Figure 3:**
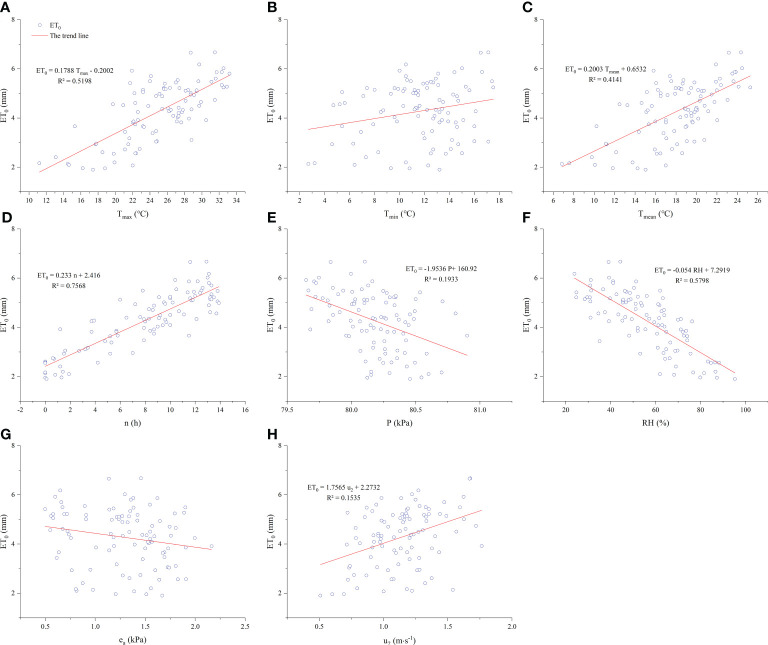
Relationships between meteorological elements and *ET*_0_ during the growing season in 2021. **(A–H)** describes the relationship between daily maximum temperature (T_max_), daily minimum temperature (T_min_), daily mean temperature (T_mean_), sunshine hours (n), atmospheric pressure (P), relative air humidity (RH), water vapour pressure (e_a_), wind speed (u_2_) and reference crop evapotranspiration (ET_0_), respectively.

**Figures 3A–H** shows the functional correlation between a particular meteorological factor and *ET*_0_ during the watermelon growing season. From **Figure 3**, we can see that *T_max_*, *T_mean_*, *n*, and *RH* were highly correlated with *ET*_0_ with an R^2^ > 0.4 (*p*< 0.05); and *P* was somewhat negatively correlated with *ET*_0_, but the R^2^ was only 0.1933. While *T_min_*, *e_a_*, and *u*_2_ did not demonstrate a functional relationship for the effect of *ET*_0_. With an R^2^ of 0.757, both *T* and *n* were significantly and positively correlated with *ET*_0_, indicating that temperature, sunshine duration, and wind speeds had a significant promoting effect on *ET*_0_.

**Figure 4:**
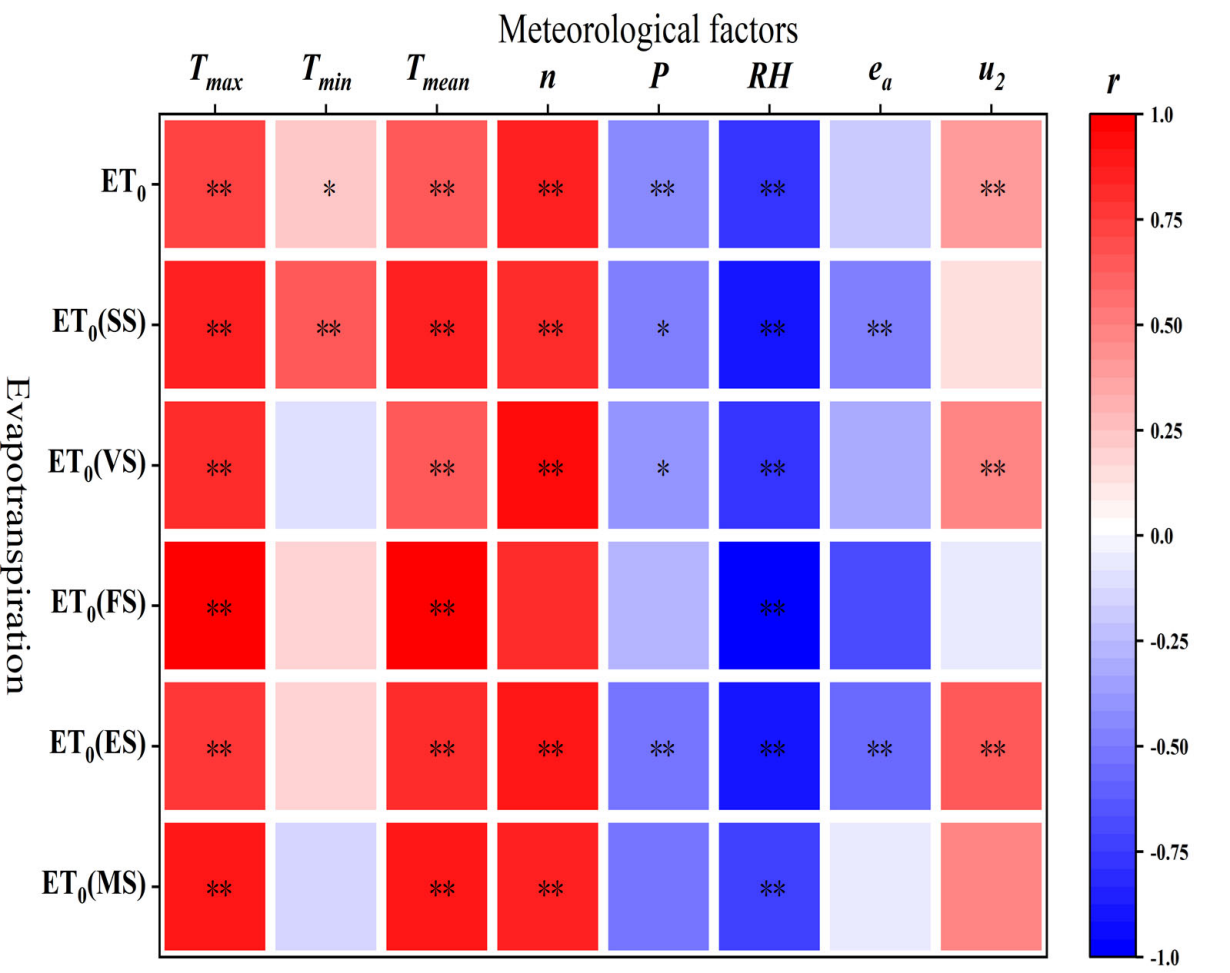
Correlation analysis of *ET*_0_ and meteorological elements during the watermelon growth period. * and ** represent significance at the p<0.05 and 0.01 levels , respectively.

The *r* and significance analysis was subsequently computed for each step to conduct a thorough investigation into the relationship between *ET*_0_ and various climatic parameters within each growth stage. The results are displayed in **Figure 4**. The correlations between meteorological factors and total *ET*_0_ during the entire growing season were consistent with the results of the above analysis. The correlations between *ET*_0_ and each meteorological factor within different growth stages had co-factor influences, although there was some variability. Both the *r* of *n* at FS and *P* at MS with *ET*_0_ were 0.823 and 0.509, but neither was statistically significant. This was mostly because the growing time was not significantly affected by the relatively small sample size. *T_min_* peaked at the SS and became extremely significant, but they were unimportant at all other growth stages and had negative correlations at the VS and MS. While *u*_2_ demonstrated a highly significant positive correlation at both the VS and ES with no significant influence at any other stage, *e_a_* displayed a highly significant negative correlation at both the SS and the ES.

To further explore the role of multiple factors in driving the dependent variable, a multiple linear regression equation was constructed between *ET*_0_ and the meteorological parameters subsumed in its calculation for the watermelon growing season and each period. **Table 4** displays the specific results, and the R^2^ was all above 0.9 (*p*< 0.01), which was a better fit.

**Table 4:** Multiple linear regression analysis of meteorological elements and *ET*_0_ in each growth stage.

The growth stages	The regression equation	R^2^
The entire growing season	*ET_0 =_* 0.152*n* + 0.772*u_2_* - 0.900*e_a_* - 0.366 + 0.095*T_min_* + 0.076*T_max_* + 29.701	0.940
SS	*ET*_0 =_ 0.143*T_max_* + 0.148*n* - 0.558*P* + 0.927*u*_2 +_ 44.533	0.943
VS	*ET*_0 =_ 0.145*n* + 0.073*T_max_* + 0.819*u*_2_ - 0.014*RH* + 1.202	0.943
FS	*ET*_0 =_ 0.593*T_mean_* - 0.203*T_min_* + 0.39*u*_2_ - 0.005*n* - 5.472	0.999
ES	*ET*_0_ = -0.024*RH* + 0.799*u*_2 +_ 0.158*n* + 0.267*T_mean_* - 0.133*T_max_* + 1.938	0.969
MS	*ET*_0 =_ 0.084*T_max_* + 0.115*n* - 0.025*RH* + 2.419	0.941

### 3.3 Effects of different WD treatments on *ET*

**Tables 5**–**9** show the *ET*, *K_c_*, *ET* intensity, and water consumption modulus of oasis watermelon under various water treatments during various growing period. The *ET* of watermelon during the whole growing season was 323 mm and 334 mm in 2020 and 2021, respectively, with adequate water supply. Compared with CK, the *ET* of WD treatments decreased to different degrees, and the most significant was the WD treatment at the ES, while the water consumption of watermelon showed a bimodal trend during the growing season.

**Table 5:** *ET*, *K_c_*, *ET* intensity, and water consumption modulus under different water treatments at the SS.

Year	Treatments	ET/mm	ET intensity/mm·d^-1^	Water consumption modulus/%	Water stress coefficient	*K_c_*
2020	CK	55.76 ± 0.73ab	1.99 ± 0.03ab	17.25	–	0.459 ± 0.006ab
	WD1	52.05 ± 0.72c	1.86 ± 0.03c	16.41	0.935	0.429 ± 0.006c
	WD2	48.60 ± 0.60d	1.74 ± 0.02d	16.14	0.871	0.400 ± 0.005d
	WD3	55.75 ± 0.49ab	1.99 ± 0.02ab	18.43	–	0.459 ± 0.004ab
	WD4	56.67 ± 1.17a	2.02 ± 0.04a	20.03	–	0.467 ± 0.01a
	WD5	55.21 ± 1.38ab	1.97 ± 0.05ab	18.87	–	0.455 ± 0.011ab
	WD6	55.52 ± 1.06ab	1.98 ± 0.04ab	19.79	–	0.458 ± 0.009ab
	WD7	56.30 ± 0.47ab	2.01 ± 0.02ab	17.57	–	0.464 ± 0.004ab
	WD8	54.98 ± 0.69b	1.96 ± 0.02b	17.29	–	0.453 ± 0.006b
2021	CK	53.50 ± 0.61ab	2.06 ± 0.02ab	16.01	–	0.473 ± 0.005ab
	WD1	50.05 ± 0.65c	1.93 ± 0.03c	15.24	0.934	0.442 ± 0.006c
	WD2	46.66 ± 0.65d	1.79 ± 0.03d	15.08	0.871	0.412 ± 0.006d
	WD3	54.02 ± 0.59a	2.08 ± 0.02a	17.45	–	0.477 ± 0.005a
	WD4	53.7 ± 0.52ab	2.07 ± 0.02ab	18.49	–	0.474 ± 0.005ab
	WD5	54.02 ± 0.67a	2.08 ± 0.03a	17.92	–	0.477 ± 0.006a
	WD6	52.75 ± 0.4b	2.03 ± 0.02b	18.14	–	0.466 ± 0.004b
	WD7	54.04 ± 0.81a	2.08 ± 0.03a	16.40	–	0.477 ± 0.007a
	WD8	53.95 ± 0.45a	2.07 ± 0.02a	16.41	–	0.477 ± 0.004a

The values shown are the mean ± SD, n = 3. Different lowercase letters on a line indicate significant differences at p< 0.05.

**Table 6:** *ET*, *K_c_*, *ET* intensity, and water consumption modulus under different water treatments at the VS.

Year	Treatments	ET/mm	ET intensity/mm·d^-1^	Water consumption modulus/%	Water stress coefficient	*K_c_*
2020	CK	87.3 ± 0.53a	2.91 ± 0.02a	27.01	–	0.68 ± 0.004a
	WD1	86.85 ± 1.14a	2.9 ± 0.04a	27.39	0.996	0.677 ± 0.009a
	WD2	81.02 ± 1.67b	2.7 ± 0.06b	26.91	0.928	0.631 ± 0.013b
	WD3	76.60 ± 0.90c	2.55 ± 0.03c	25.33	0.878	0.597 ± 0.007c
	WD4	70.58 ± 0.90d	2.35 ± 0.03d	24.95	0.809	0.550 ± 0.007d
	WD5	86.36 ± 0.73a	2.88 ± 0.02a	29.52	–	0.673 ± 0.006a
	WD6	86.25 ± 1.12a	2.88 ± 0.04a	30.75	–	0.672 ± 0.009a
	WD7	86.82 ± 0.18a	2.89 ± 0.01a	27.09	–	0.677 ± 0.001a
	WD8	87.21 ± 0.90a	2.91 ± 0.03a	27.42	–	0.68 ± 0.007a
2021	CK	90.29 ± 1.2a	3.01 ± 0.04a	27.00	–	0.771 ± 0.01a
	WD1	89.16 ± 0.91a	2.97 ± 0.03a	27.15	0.987	0.761 ± 0.008a
	WD2	82.73 ± 1.17b	2.76 ± 0.04b	26.74	0.916	0.706 ± 0.010b
	WD3	78.49 ± 0.86c	2.62 ± 0.03c	25.36	0.869	0.670 ± 0.007c
	WD4	73.26 ± 0.48d	2.44 ± 0.02d	25.23	0.811	0.625 ± 0.004d
	WD5	88.97 ± 1.31a	2.97 ± 0.04a	29.51	–	0.759 ± 0.011a
	WD6	88.68 ± 1.35a	2.96 ± 0.05a	30.50	–	0.757 ± 0.012a
	WD7	88.9 ± 0.67a	2.96 ± 0.02a	26.98	–	0.759 ± 0.006a
	WD8	89.79 ± 0.39a	2.99 ± 0.01a	27.32	–	0.766 ± 0.003a

The values shown are the mean ± SD, n = 3. Different lowercase letters on a line indicate significant differences at p< 0.05.

**Table 7:** *ET*, *K_c_*, *ET* intensity, and water consumption modulus under different water treatments at the FS.

Year	Treatments	ET/mm	ET intensity/mm·d^-1^	Water consumption modulus/%	Water stress coefficient	*K_c_*
2020	CK	22.27 ± 0.39a	4.45 ± 0.08a	6.89	–	1.154 ± 0.02a
	WD1	22.06 ± 0.31a	4.41 ± 0.06a	6.96	0.990	1.143 ± 0.016a
	WD2	20.74 ± 0.34b	4.15 ± 0.07b	6.89	0.932	1.075 ± 0.018b
	WD3	19.74 ± 0.13c	3.95 ± 0.03c	6.53	0.886	1.023 ± 0.007c
	WD4	18.33 ± 0.57d	3.67 ± 0.11d	6.48	0.823	0.950 ± 0.029d
	WD5	22.54 ± 0.41a	4.51 ± 0.08a	7.71	–	1.168 ± 0.021a
	WD6	22.34 ± 0.28a	4.47 ± 0.06a	7.97	–	1.158 ± 0.015a
	WD7	22.08 ± 0.2a	4.42 ± 0.04a	6.89	–	1.144 ± 0.011a
	WD8	22.21 ± 0.32a	4.44 ± 0.06a	6.98	–	1.151 ± 0.017a
2021	CK	23.41 ± 0.28a	4.68 ± 0.06a	7.00	–	1.006 ± 0.012a
	WD1	23.27 ± 0.37a	4.65 ± 0.07a	7.09	0.994	1.000 ± 0.016a
	WD2	21.66 ± 0.1b	4.33 ± 0.02b	7.00	0.925	0.931 ± 0.004b
	WD3	20.56 ± 0.12c	4.11 ± 0.02c	6.64	0.878	0.883 ± 0.005c
	WD4	19.18 ± 0.31d	3.84 ± 0.06d	6.61	0.819	0.824 ± 0.013d
	WD5	23.16 ± 0.03a	4.63 ± 0.01a	7.68	–	0.995 ± 0.001a
	WD6	23.35 ± 0.52a	4.67 ± 0.1a	8.03	–	1.003 ± 0.022a
	WD7	23.08 ± 0.48a	4.62 ± 0.1a	7.00	–	0.991 ± 0.021a
	WD8	23.25 ± 0.29a	4.65 ± 0.06a	7.07	–	0.999 ± 0.012a

The values shown are the mean ± SD, n = 3. Different lowercase letters on a line indicate significant differences at p< 0.05.

**Table 8:** *ET*, *K_c_*, *ET* intensity, and water consumption modulus under different water treatments at the ES.

Year	Treatments	ET/mm	ET intensity/mm·d^-1^	Water consumption modulus/%		Water stress coefficient	*K_c_*
2020	CK	125.61 ± 0.69a	5.23 ± 0.03a		38.86	–	1.246 ± 0.007a
	WD1	124.16 ± 3.16ab	5.17 ± 0.13ab		39.14	0.989	1.232 ± 0.031ab
	WD2	119.04 ± 5.27bc	4.96 ± 0.22bc		39.52	0.948	1.181 ± 0.052bc
	WD3	118.7 ± 0.74c	4.95 ± 0.03c		39.25	0.945	1.178 ± 0.007c
	WD4	107.21 ± 2.13d	4.47 ± 0.09d		37.89	0.854	1.064 ± 0.021d
	WD5	101.87 ± 4.74e	4.24 ± 0.2e		34.80	0.811	1.011 ± 0.047e
	WD6	91.75 ± 1.54f	3.82 ± 0.06f		32.71	0.730	0.910 ± 0.015f
	WD7	124.79 ± 2.22a	5.20 ± 0.09a		38.94	–	1.238 ± 0.022a
	WD8	125.68 ± 2.97a	5.24 ± 0.12a		39.51	–	1.247 ± 0.029a
2021	CK	133.76 ± 6.34a	5.82 ± 0.28a		39.99	–	1.237 ± 0.059a
	WD1	132.66 ± 1.74a	5.77 ± 0.08a		40.39	0.992	1.227 ± 0.016a
	WD2	125.37 ± 1.72b	5.45 ± 0.07b		40.52	0.938	1.160 ± 0.016b
	WD3	123.78 ± 0.80b	5.38 ± 0.03b		40.00	0.926	1.145 ± 0.007b
	WD4	113.24 ± 1.18c	4.92 ± 0.05c		39.00	0.847	1.048 ± 0.011c
	WD5	108.14 ± 1.12d	4.70 ± 0.05d		35.86	0.808	1.000 ± 0.01d
	WD6	99.65 ± 0.42e	4.33 ± 0.02e		34.27	0.745	0.922 ± 0.004e
	WD7	131.89 ± 0.49a	5.73 ± 0.02a		40.03	–	1.220 ± 0.005a
	WD8	132.68 ± 0.28a	5.77 ± 0.01a		40.36	–	1.227 ± 0.003a

The values shown are the mean ± SD, n = 3. Different lowercase letters on a line indicate significant differences at p< 0.05.

**Table 9:** *ET*, *K_c_*, *ET* intensity, and water consumption modulus under different water treatments at the MS.

Year	Treatments	ET/mm	ET intensity/mm·d^-1^	Water consumption modulus/%	Water stress coefficient	*K_c_*
2020	CK	32.29 ± 0.99a	2.94 ± 0.09a	9.99	–	0.724 ± 0.022a
	WD1	32.05 ± 0.32a	2.91 ± 0.03a	10.11	0.992	0.718 ± 0.007a
	WD2	31.73 ± 0.62a	2.88 ± 0.06a	10.54	0.982	0.711 ± 0.014a
	WD3	31.63 ± 0.36a	2.88 ± 0.03a	10.46	0.979	0.709 ± 0.008a
	WD4	30.13 ± 0.44b	2.74 ± 0.04b	10.65	0.932	0.675 ± 0.01b
	WD5	26.64 ± 0.66d	2.42 ± 0.06d	9.10	0.825	0.597 ± 0.015d
	WD6	24.63 ± 0.66e	2.24 ± 0.06e	8.78	0.762	0.552 ± 0.015e
	WD7	30.46 ± 0.37b	2.77 ± 0.03b	9.50	0.943	0.683 ± 0.008b
	WD8	28.01 ± 0.39c	2.55 ± 0.04c	8.80	0.867	0.628 ± 0.009c
2021	CK	33.44 ± 0.55a	2.79 ± 0.05a	10.00	–	0.688 ± 0.011a
	WD1	33.29 ± 0.58a	2.77 ± 0.05a	10.13	0.996	0.685 ± 0.012a
	WD2	32.98 ± 0.06a	2.75 ± 0.01a	10.66	0.985	0.678 ± 0.001a
	WD3	32.64 ± 0.73a	2.72 ± 0.06a	10.55	0.975	0.671 ± 0.015a
	WD4	30.96 ± 0.44b	2.58 ± 0.04b	10.66	0.926	0.637 ± 0.009c
	WD5	27.24 ± 0.55d	2.27 ± 0.05d	9.03	0.814	0.56 ± 0.011d
	WD6	26.32 ± 0.19e	2.19 ± 0.02e	9.05	0.786	0.541 ± 0.004e
	WD7	31.59 ± 0.53b	2.63 ± 0.04b	9.59	0.945	0.650 ± 0.011b
	WD8	29.05 ± 0.72c	2.42 ± 0.06c	8.84	0.869	0.598 ± 0.015c

The values shown are the mean ± SD, n = 3. Different lowercase letters on a line indicate significant differences at p< 0.05.

#### 3.3.1 Effects of WD at the seedling stage (SS)

**Table 5** shows that for mild (WD1) and moderate (WD2) WD, respectively, the *ET* was considerably reduced in 2020 and 2021 compared with CK by 6.45%–6.65% and 12.79%–12.84%, respectively (*p*< 0.05), whereas the other treatments did not significantly differ from CK (*p* > 0.05). The *ET* intensities of WD1 and WD2 were 1.85–1.93 mm·d^-1^ and 1.74–1.79 mm·d^-1^, respectively, and both were significantly lower than CK. The moderate treatment saw a higher decline, whereas the other treatments did not differ significantly from CK. The water consumption modulus of the treatments ranged from 15.08%–20.03% in both years due to short, slow-growing watermelon plants and relatively low temperatures during the SS.

#### 3.3.2 Effect of WD at the vine stage (VS)

As can be seen from **Table 6**, ET was highest in 2020 with 87.30 mm for CK, and 89.79 mm for treatment WD8 in 2021, which was only 2.85% higher than that of CK, but the difference was not significant, while *ET* was lowest in 2a moderate WD treatment WD4 with a decrease of 16.08%–19.15%, while mild WD treatment WD3 decreased by 10.09%–12.26%, and the rehydration treatment WD1 was at the same level as CK. The *ET* intensity was highest in CK, with 2a at 2.91 mm·d^-1^ and 3.01 mm·d^-1^ respectively, followed by the rehydration treatment WD1. The ET intensity of treatment WD4 was significantly reduced by 18.85%–19.15% compared to CK, and the reduction in treatment WD3 was 12.26%–13.07%. When watermelon entered the VS, the root crown developed rapidly, the temperature rose gradually and the water consumption modulus increased, which ranged from 24.95% to 30.75%.

#### 3.3.3 Effect of WD at the flowering and fruiting (FS)

**Table 7** indicates that the water consumption of the various water treatments was the same as that of the preceding stage, with a relatively small water consumption modulus of 6.48%–8.03%. This was caused by the stage’s brief duration as well as the stage’s higher *ET* intensity of approximately 4.30 mm·d^-1^, which occurred as the plants were in a crucial stage of transition from nutritional to reproductive growth.

#### 3.3.4 Effect of WD at the expansion stage (ES)

As can be seen from **Table 8**, *ET* was highest for CK in 2020 and 2021, with treatments WD1 and WD3 not significantly different from CK, while *ET* for the mild (WD5) and moderate (WD6) WD treatments decreased significantly by 18.90% and 26.95%, respectively, compared to CK, while WD treatments WD3 and WD4 were still smaller than CK during the VS, and the differences were significant. The *ET* intensity was highest in CK, reaching 5.23 mm·d^-1^ and 5.82 mm·d^-1^, but the effect of WD on watermelon water consumption was greater at this stage, with treatments WD5 decreasing by 18.93%–19.24%, while treatment WD6 had the least *ET* intensity, decreasing by 25.60%–26.96%. Since the ES was the critical period of water demand for watermelon, to ensure the rapid expansion of the flesh cells, water demand was at its maximum, with a water consumption modulus of 32.71%–41.18%.

#### 3.3.5 Effect of WD at the maturity stage (MS)

As can be seen from **Table 9**, in 2020 and 2021, evapotranspiration was highest in CK, while treatment WD6 remained the lowest at 24.63 mm and 26.32 mm, significantly lower than CK by 23.72% and 21.29%, respectively, while treatments WD2 and WD3 were not significantly different from CK, while mild (WD7) and moderate (WD8) WD treatments were significantly lower than CK by 5.53%–5.67% and 13.13%–13.25%, respectively. The evapotranspiration intensity of CK was still the highest, with treatments WD1–WD3 at the same level as CK, but WD8 demonstrated a significant decrease of 13.26%–18.45% compared with CK, and WD7 indicated a decrease of 5.67%–2.73%. Moreover, at watermelon MS, the growth rate also slowed down and the water consumption modulus dropped to 8.78%–10.66%.

In summary, (1) the *ET* results revealed that the *ET* of the SS in 2020 was similar to that of the SS in 2021, and the remaining treatments except for the WD at this stage were not significantly different from CK. The *ET* at the VS and ES decreased with increasing WD levels, with a significant decrease compared with CK, and the *ET* at the ES in 2021 was higher than that in 2020. The *ET* of CK was the highest at the MS. (2) The *ET* intensity showed that it was lowest at the SS (about 1.94 mm·d^-1^), higher at the VS and MS (about 2.81 and 2.59 mm·d^-1^), and highest at the FS (about 4.36 and 5.14 mm·d^-1^). The *ET* intensity of oasis watermelon ranged from 1.74 to 5.82 m·d^-1^ under different water treatments throughout the entire reproductive period. (3) The water consumption modulus showed a relatively similar distribution pattern for watermelon during the two-year reproductive period, i.e., 32.71%–40.52% at the ES, 24.95%–30.75% at the VS, 15.24%–20.03% at the SS and 8.78%–10.66% at the MS. This is mainly since many factors influence the water consumption modulus, such as *ET* intensity, total evapotranspiration during the growing season, environmental factors, and the duration of the growth stage, which cannot be directly related to the magnitude of the WD.

The *K_c_* is related to the crop type, variety, reproductive period, and *LAI*, reflecting the physiological characteristics of the crop itself. As can be seen from **Tables 5**-**9**, *K_c_* generally showed a single-peaked curve fluctuation of increasing and then decreasing, reaching peaks of 1.246 and 1.237 at the ES, followed by 1.154 and 1.006 at the FS, and a minimum of 0.459 and 0.473 at the SS. Irrigation had a large effect on *K_c_*, with any stage of WD reducing the *K_c_* for that stage, and the greater the stress the smaller the corresponding *K_c_*. *K_c_* for both years was smallest at 0.400 and 0.412 for the moderate WD treatment at SS (WD2), largest in 2020 (1.247), and largest in 2021 (1.237) for the moderate WD treatment at ES (WD8), while the average values of *K_c_* were 0.803 and 0.784 for the whole reproductive period.

### 3.4 Effects of different WD treatments on *LAI*

**Figure 5:**
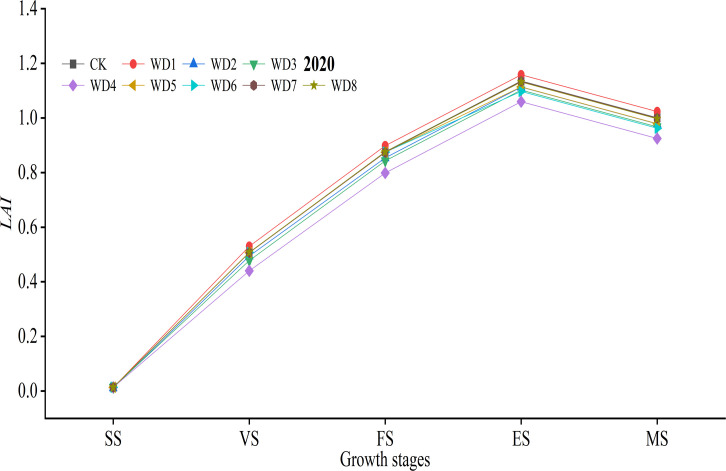
*LAI* curve with time for different water treatments.

**Figure 5** shows the variation curves of watermelon *LAI* with time under different water treatments. The trend of the *LAI* of oasis watermelon under different water treatments is generally similar, with the *LAI* gradually increasing from the beginning of the growing season, and the *LAI* increasing rapidly during the VG, which is a critical stage for the growth of watermelon vine leaves and other nutrient organs, reaching a peak in the early ES. The late ES to the MS, the plant’s nutritional growth stalled, most of the vine-leaves nutrients were absorbed by the fruit, the crop metabolism tended to slow down, the base leaves gradually withered and fell off, and the *LAI* showed a slow decrease trend.

For reasons of length, only 2020 data was analyzed. Compared with CK, WD treatments WD1 and WD2 at SS reduced *LAI* by 6.35% and 16.90%, respectively, with significant differences (*p*< 0.05). At VS, WD1 grew faster and higher than CK by 4.42% after rehydration, but the difference was not significant (*p* > 0.05), while WD2 still decreased by 2.58%, and the *LAI* of WD treatments WD3 and WD4 decreased significantly by 5.80% and 13.25%, respectively, compared with CK. The results of all treatments at FS were the same as that of VS. Upon entering the ES, the *LAI* of treatment WD1 was the largest, while the leaf growth of treatments WD2 and WD3 accelerated after rehydration and were still lower than that of CK but not significantly different, and the WD treatments WD5 and WD6 were both lower than that of CK during this period, with a significant difference for treatment WD6. By the end of MS, the *LAI* of all treatments decreased, among which all treatments were lower than CK except for the mild WD treatment at the SS, with a decrease of 0.11%–7.53%, while treatment WD4 (moderate WD at the VS) was significantly different from CK. The *LAI* was affected differently by different WD treatments at different growth stages. SS to the beginning of FS was the rapid formation stage of watermelon stems and leaves and other nutrient organs, WD at this stage had a significant effect on the *LAI*, but it is not significantly different from CK due to the compensation result of light stress after rehydration.

### 3.5 Relationship between *K_c_* and *LAI*

**Figure 6:**
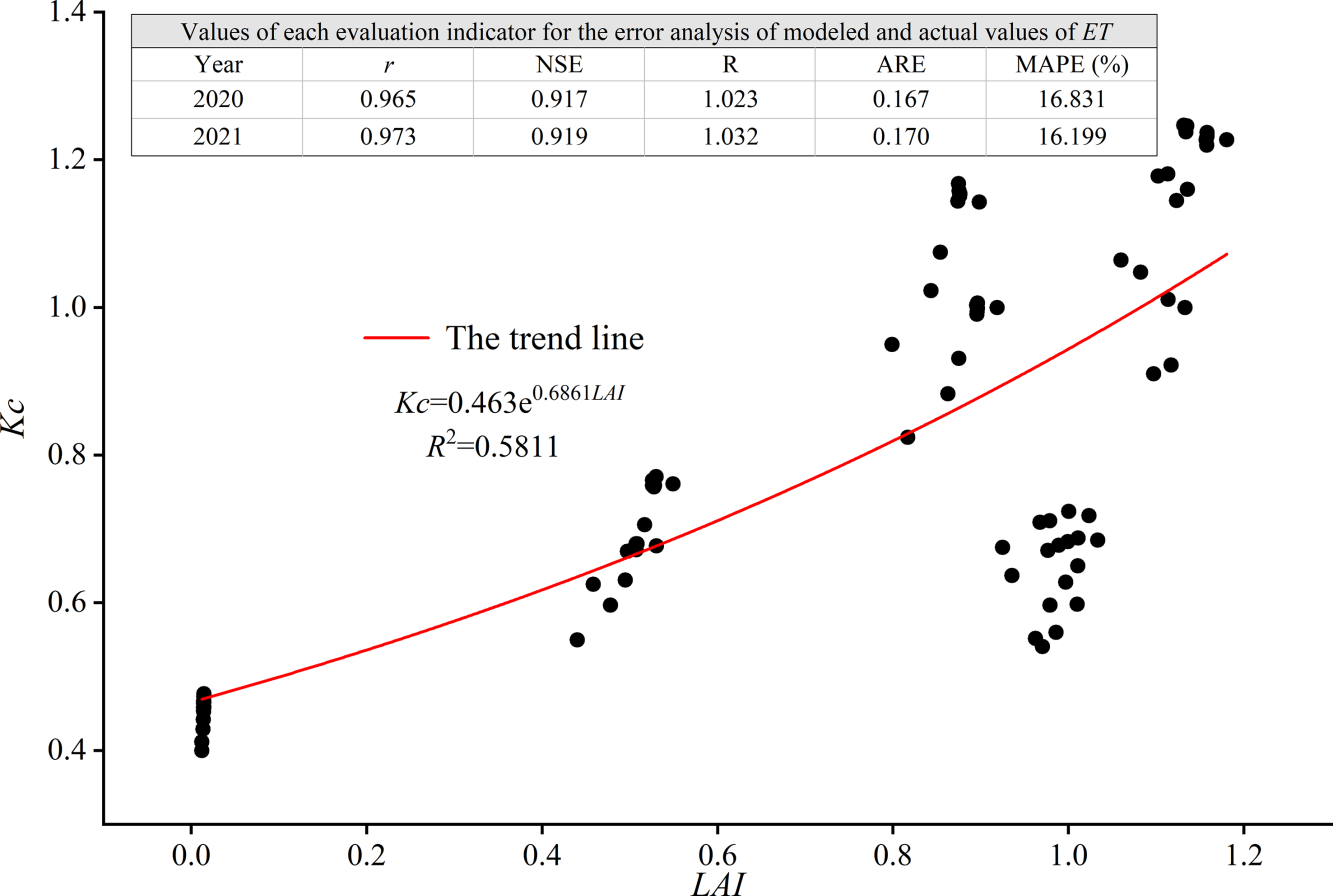
Relationship between *K_c_* and *LAI* for oasis watermelon.

*K_c_* has a dynamic process of change during the reproductive period, and it is generally accepted that there is a correlation between *K_c_* and *LAI*. In this paper, the regression equation between *K_c_* and mean *LAI* under different water treatments was established based on the actual data measured in two consecutive growing seasons of oasis watermelon in the Hexi region, as shown in **Figure 6**.

The *LAI* and *K_c_* were first analyzed for correlation, with *r* = 0.759 (*p*< 0.01), indicating a highly significant positive correlation between the *LAI* and *K_c_* of oasis watermelon. The regression analysis in **Figure 6** shows that there was a good exponential relationship between *K_c_* and *LAI* for oasis watermelon, with the fitted relationship equation ${\text{K}}_{\text{c}}=0{.463\text{e}}^{0.686\text{LAI}}$ and R^2^ = 0.5811 (*p*< 0.01), indicating that it is possible to estimate the *K_c_* by measuring the *LAI* of watermelon using this relationship equation, thereby providing a practical reference for watermelon irrigation.

### 3.6 *ET* estimation model and error analysis

The quantitative relationship between the *LAI* and *K_c_* and the integrated P-M equation enabled the modeling of the actual *ET* of oasis watermelon in the Hexi region with the *LAI* and meteorological factors.


(16) $$ET=ET_{0}\times K_{c}=ET_{0}\times 0.463e^{0.6861LAI}$$

To evaluate the reliability of the model established under different water treatment conditions, the experimental data from 2020 and 2021 were employed to validate the established *ET* estimation model. The quantitative evaluation analysis was conducted by comparing the actual evapotranspiration values (*ET_s_*) obtained from the water balance equation with the modeled values (*ET_m_*). The evaluation results are shown in **Figure 6**.

**Figure 6** shows the results of the statistical coefficients comparing *ET_s_* with *ET_m_* for the assessment phase of the model equation. The *r* for the actual values obtained from the water balance method and the estimated model modeling values in 2020 and 2021 were 0.965 and 0.973 respectively (*p*< 0.01), i.e., the correlation between both was good; the NSE was 0.917 and 0.919 respectively, i.e., the NSH of the model constructed was close to 1, indicating that the model had better fitting accuracy and high reliability; the R was 1.023 and 1.032 respectively, i.e., the totals were similar, which showed that the model has a better performance in the evaluation phase. In addition, ARE values for two-year were small and the MAPE values were 16.831% and 16.199%, indicating that the errors in the modeling results were relatively small. It can be shown that the model constructed in this study can better estimate the watermelon *ET* under the conditions of sub-membrane drip irrigation in the Hexi Oasis by using *LAI* and meteorological factors.

## 4 Discussion

### 4.1 Variations of *ET*_0_ during the watermelon growing season

*ET*_0_ is a critical parameter for calculating crop water requirements and irrigation management (Ahmad et al., 2017; Kadam et al., 2021), and the *ET*_0_ calculated based on the P-M equation is directly affected by the local climate (Neto et al., 2015; Borges and Pinheiro, 2019). In this study, the average *ET*_0_ values during the watermelon growing season were found to be 4.15 mm (2020) and 4.33 mm (2021), with the relatively smallest *ET*_0_ at MS. Simple correlation analysis between meteorological factors and *ET*_0_ during the growing period showed that *ET*_0_ was positively correlated with *T*, *n*, and *u*_2_, while it was negatively correlated with *P*, *RH*, and *e_a_*. Similar findings were obtained by Zeng (2018), which were mainly related to the evaporative properties of water. To further clarify the influence of meteorological factors on *ET*_0_, a multiple linear regression model was established between *ET*_0_ and the meteorological factors involved in the calculation for the whole reproductive period and stage, and the fitting results were high. Wang et al. (2014) achieved similar results for the *ET*_0_ estimation in the Hetao areas.

### 4.2 The *ET* pattern of oasis watermelon

*ET*_0_ is determined by soil, irrigation, crop varieties, and meteorology, but WD can effectively reduce *ET* of melon crops (Leite et al., 2015; Qin and Leskovar, 2020), especially at the fruit growth and development stage, which has a more significant effect (Yavuz et al., 2021). According to the water consumption of the two-year, WD at the VS and ES had a greater impact on the stage *ET* of watermelon, thereby leading to a significant reduction in total *ET*. Compared with CK, stage *ET* was significantly reduced by 10.09%–1.32% for mild WD at VS (WD3), and by 16.08%–19.15% for moderate WD treatment (WD4), and the difference between WD3 and WD4 was significant. During the ES, stage water consumption was highest in CK at 125.61 mm in 2020 and 133.76 mm in 2021, and was significantly reduced by 18.90%–19.16% and 25.50%–26.95% in the mild (WD5) and moderate (WD6) WD treatments, respectively, during this period. The difference between them was also significant. This is consistent with the findings of Zhang et al. (2021), where stage WD resulted in a linear decrease in the average daily *ET* of watermelon, with the greatest decrease occurring during the ES.

Studies (Ji et al., 2015) have shown that water regulation effectively reduced the stage water consumption modulus and *ET* intensity of crops. The results of two consecutive growing seasons indicated that *ET* showed an increasing and then decreasing law, with the highest water consumption modulus (about 40%) at the ES, followed by the second highest (29%) at the VS, the smallest (about 7%) at the FS, and about 19% and 10% at the SS and MS. The *ET* intensity is the smallest at the SS (about 1.9 mm·d^-1^), relatively large at the VS and MS (about 2.8 mm·d^-1^), and the largest at the FS (4–5 mm·d^-1^). This is because crop water requirements are mainly influenced by growth characteristics, meteorology, and agronomic management practices, but also follow the corresponding habitual and quantitative patterns. Furthermore, WD at various growth stages caused a decrease in both water consumption modulus and *ET* intensity of watermelon, and the more the corresponding stage *ET*, the more significant the effect of WD-induced decrease in water consumption. This is similar to the results of Wang et al. (2018b) and Huang et al. (2021) on the water requirements patterns of WD peppers.

### 4.3 Relationship between *LAI* and *K_c_*

*K_c_* can be employed to describe the effect of crop biological features, water productivity, and soil tillage conditions on crop water requirements as an indirect parameter for determining *ET*_0_ (Wright, 1982; Pereira et al., 1999). Related studies (Yun et al., 2015; Li et al., 2022b) revealed that between crop *LAI* and *K_c_* existed a more favorable exponential relationship, which was consistent with the results of this work but differed from the findings of Hu et al. (2012) and He and Wu (2018), who discovered a quadratic curve relationship between *K_c_* and *LAI* of drip irrigated jujube trees. This might be related to crop varieties and cultivation areas. In addition, this paper established an *ET* estimation model for watermelon with under-mulched drip irrigation in the Hexi region based on the above-mentioned exponential regression relationship, and after evaluation and validation, the NSE reached more than 0.9, which was higher than the NSE of *ET* models established in different approaches by Wang et al. (2018a), thereby filling a research gap in the estimation of *K_c_* and *ET* for oasis watermelon.

## 5 Conclusion

(1) Water deficit at various growth stages led to a decrease in the evapotranspiration intensity of watermelon. Evapotranspiration was relatively highest at the ES, which is a critical water requirement period for oasis watermelon, ranging from 91.75 to 133.76 mm with an average evapotranspiration intensity of 5.06 mm·d^-1^.

(2) There was a promotion of ET_0_ by temperature, hours of sunlight, and wind speed, and the potential impact of high-temperature variation on watermelon growth was greater than other climatic factors. The crop coefficients for field watermelon at the SS, VS, FS, ES, and MS were in the order of 0.400–0.477, 0.550–0.771, 0.824–1.168, 0.910–1.237, and 0.552–0.803, under water deficit conditions in Hexi region.

(3) The leaf area index varied more significantly among the different water treatments and increased with increasing irrigation. Moreover, there was a strongly significant correlation between crop coefficient and leaf area index, showing a well-exponential relationship. Based on this relationship, a model for estimating the evapotranspiration of oasis watermelons concerning the leaf area index was also established, and the modeling results of the proposed model were empirically analyzed to be in good agreement with the actual values.

The model in this work is based on the results of two consecutive growing seasons trials of water deficit watermelon, and only single crop coefficient method were used to calculate and establish the estimation model, which might have some bias. In future studies, we will combine years of actual measurement data to build an evapotranspiration estimation model according to the dual-crop coefficient method to further improve the accuracy and practical application of the model.

## Data availability statement

The original contributions presented in the study are included in the article/**Supplementary Material**. Further inquiries can be directed to the corresponding author.

## Author contributions

ZW and HZ prepared the experimental scheme. ZW prepared data analysis and drafted the article. HZ was responsible for the funding acquisition. HZ and SY revised the experimental protocol and article format. ZW, AT, CZ, LL, YB, and XC performed part of the experiments and provided some of the experimental results for the manuscript. All authors contributed to the article and approved the submitted version.

## References

[B1] Ahmad L. Parvaze S. Parvaze S. Kanth R. H. (2017). FAO reference evapotranspiration and crop water requirement of apple (Malus pumila) in Kashmir valley. J. Agrometeorol. 19, 262–264. doi: 10.54386/jam.v19i3.668

[B2] Allen R. G. Jensen M. E. Wright J. L. Burman R. D. (1989). Operational estimates of reference evapotranspiration. Agron. J. 81, 650–662. doi: 10.2134/agronj1989.00021962008100040019x

[B3] Allen R. G. Pereira L. S. Howell T. A. Jensen M. E. (2011). Evapotranspiration information reporting: i. factors governing measurement accuracy. Agric. Water Manage. 98, 899–920. doi: 10.1016/j.agwat.2010.12.015

[B4] Allen R. G. Pereira L. S. Raes D. Smith M. (1998). Crop evapotranspiration-guidelines for computing crop water requirements-FAO irrigation and drainage paper 56 (Rome: Food and Agriculture Organization of the United Nations), D05109.

[B5] Borges J. C. F. Pinheiro M. A. B. (2019). Daily reference evapotranspiration based on temperature for brazilian meteorological stations. J. Irrig. Drain. Eng. 145, 04019029. doi: 10.1061/(ASCE)IR.1943-4774.0001437

[B6] Chai Q. Gan Y. Turner N. C. Zhang R. Yang C. Niu Y.. (2014). Water-saving innovations in Chinese agriculture. Adv. Agron. 126, 149–201. doi: 10.1016/B978-0-12-800132-5.00002-X

[B7] Cui N. Du T. Kang S. Li F. Zhang J. Wang M.. (2008a). Regulated deficit irrigation improved fruit quality and water use efficiency of pear-jujube trees. Agric. Water Manage. 95, 489–497. doi: 10.1016/j.agwat.2007.11.007

[B8] Cui X. Xue C. Yang X. Yang J. Zhang Q. Bouman B. A. M. (2008b). Field evapotranspiration characteristics and water use efficiency of aerobic rice under different water treatments. trans. chin. Soc Agric. Eng. 24, 49–54. doi: 10.3321/j.issn:1002-6819.2008.04.009

[B9] Deng Y. Gou X. Gao L. Yang M. Zhang F. (2017). Tree-ring recorded moisture variations over the past millennium in the hexi corridor, northwest China. Environ. Earth Sci. 76, 1–9. doi: 10.1016/j.agwat.2022.107983

[B10] Du S. Kang S. Li F. Du T. (2017). Water use efficiency is improved by alternate partial root-zone irrigation of apple in arid northwest China. Agric. Water Manage. 179, 184–192. doi: 10.1016/j.agwat.2016.05.011

[B11] Feng H. Chen J. Zheng X. Xue J. Miao C. Du Q.. (2018). Effect of sand mulches of different particle sizes on soil evaporation during the freeze–thaw period. Water 10, 536. doi: 10.3390/w10050536

[B12] Glasbey C. A. Allcroft D. J. (2008). A spatiotemporal auto-regressive moving average model for solar radiation. J. R. Stat. Soc C-Appl. 57, 343–355. doi: 10.1111/j.1467-9876.2007.00617.x

[B13] He J. Cai H. Bai J. (2013). Irrigation scheduling based on CERES-wheat model for spring wheat production in the minqin oasis in Northwest China. Agric. Water Manage. 128, 19–31. doi: 10.1016/j.agwat.2013.06.010

[B14] He Z. Wu M. (2018). Crop coefficient and water consumption rule of jujube trees in bubbled-root irrigation of loess plateau region. J. Irrig. Drain. 37, 5–9. doi: 10.13522/j.cnki.ggps.20180249

[B15] Hu Y. Li Y. Zhang Y. (2012). Experiment on crop coefficient and water requirement of drip-irrigation jujube in loess plateau of china. trans. chin. Soc Agric. Mach. 43, 87–91+79. doi: 10.6041/j.issn.1000-1298.2012.11.016

[B16] Huang S. Feng Q. Lu Z. Wen X. Deo R. (2017). Trend analysis of water poverty index for assessment of water stress and water management polices: a case study in the hexi corridor China. Sustainability 9, 756. doi: 10.3390/su9050756

[B17] Huang H. Han D. Chen N. Huang D. Zhang Z. Zhang K. (2021). Water consumption pattern of capsicum annuum under regulated deficit irrigation in desert oasis. J. Natural Resour. 27, 747–756. doi: 10.11849/zrzyxb.2012.05.004

[B18] Ji X. Cheng Z. Zhang R. Zhao X. (2015). Experimental research on drip irrigation by water controlling for wine grape in arid desert oasis. Agric. Res. Arid Areas 33, 135–140. doi: 10.16302/j.cnki.1000-7601.2015.02.022

[B19] Juhasz A. Hrotko K. (2014). Comparison of the transpiration part of two sources evapotranspiration model and the measurements of sap flow in the estimation of the transpiration of sweet cherry orchards. Agric. Water Manage. 143, 142–150. doi: 10.1016/j.agwat.2014.06.014

[B20] Kadam S. A. Gorantiwar S. D. Mandre N. P. Tale D. P. (2021). Crop coefficient for potato crop evapotranspiration estimation by field water balance method in semi-arid region, maharashtra, India. Potato Res. 64, 421–433. doi: 10.1007/s11540-020-09484-8

[B21] Kuscu H. Turhan A. Ozmen N. Aydinol P. Buyukcangaz H. Demir A. O. (2015). Deficit irrigation effects on watermelon (*Citrullus vulgaris*) in a sub humid environment. J. Anim. Plant Sci. 25, 1652–1659. doi: 10.3969/10.1016/0042-207X(72)91189-X

[B22] Leite K. N. Cabello M. J. Valnir M. Tarjuelo J. M. Dominguez A. (2015). Modelling sustainable salt water management under deficit irrigation conditions for melon in Spain and Brazil. J. Sci. Food Agric. 95, 2307–2318. doi: 10.1002/jsfa.6951 25296534

[B23] Leskovar D. I. Bang H. Kim S. L. Yoo K. S. King S. R. Crosby K. (2007). Environmental and genetic factors on carotenoids and quality in watermelon fruits. Acta Hortic. 744, 233–241. doi: 10.17660/ActaHortic.2007.744.24

[B24] Li Z. Fei L. Yin Y. Li Z. Liu T. Hao K.. (2022b). Crop coefficient and evapotranspiration estimation of apple in northern shaanxi under surge-root irrigation. J. Water Resour. Water Eng. 33, 209–215. doi: 10.11705/j.issn.1672-643x.2022.02.28

[B25] Li X. Zhang H. Li F. Deng H. Wang Z. Chen X. (2022a). Evaluating effects of regulated deficit irrigation under mulched on yield and quality of pumpkin in a cold and arid climate. Water 14, 1563. doi: 10.3390/w14101563

[B26] Li X. Zhang X. Niu J. Tong L. Kang S. Du T.. (2016). Irrigation water productivity is more influenced by agronomic practice factors than by climatic factors in hexi corridor, Northwest China. Sci. Rep. 6, 37971. doi: 10.1038/srep37971 27905483 PMC5131340

[B27] Lin J. Cai H. Zheng J. Wang J. (2010). Study on high-yielding indicators of greenhouse mini-watermelon under deficit irrigation. Agric. Res. Arid Areas 28, 127–131. doi: 10.7606/j.issn.1000-7601.2010.02.26

[B28] Liu L. Mo Y. Yang X. Li X. Wu M. Zhang X.. (2014). Reasonable drip irrigation frequency improving watermelon yield and quality under regulated deficit irrigation in plastic greenhouse. Trans. Chin. Soc Agric. Eng. 30, 95–104. doi: 10.3969/j.issn.1002-6819.2014.24.012

[B29] Liu Y. Wang L. Ni G. Cong Z. (2009). Spatial distribution characteristics of irrigation water requirement for main crops in China. Trans. Chin. Soc Agric. Eng. 25, 6–12. doi: 10.3969/j.issn.1002-6819.2009.12.002

[B30] Liu C. Zhang X. Zhang Y. (2002). Determination of daily evaporation and evapotranspiration of winter wheat and maize by large-scale weighing lysimeter and micro-lysimeter. Agric. For. Meteorol. 111, 109–120. doi: 10.1016/S0168-1923(02)00015-1

[B31] Meng Q. (2011). Soil moisture consumption pattern and growth response of hilly apple orchard in the loess plate (Shangxi: Northwest A & F University).

[B32] Munitz S. Schwartz A. Netzer Y. (2019). Water consumption, crop coefficient and leaf area relations of a vitis vinifera cv. ‘Cabernet sauvignon’ vineyard. Agric. Water Manage. 219, 86–94. doi: 10.1016/j.agwat.2019.03.051

[B33] Neto A. J. S. Borges J. C. F. Andrade C. L. T. Lopes D. C. Nascimento P. T. (2015). Reference evapotranspiration estimates based on minimum meteorological variable requirements of historical weather data Chilean. J. Agric. Res. 75, 366–374. doi: 10.4067/S0718-58392015000400014

[B34] Netzer Y. Yao C. Shenker M. Bravdo B. A. Schwartz A. (2009). Water use and the development of seasonal crop coefficients for superior seedless grapevines trained to an open-gable trellis system. Irrig. Sci. 27, 109–120. doi: 10.1007/s00271-008-0124-1

[B35] Pereira L. S. Perrier A. Allen R. G. Alves I. (1999). Evapotranspiration: concepts and future trends. J. Irrig. Drain. Eng. 125, 45–51. doi: 10.1061/(asce)0733-9437(1999)125:2(45

[B36] Qin K. Leskovar D. I. (2020). Assessments of humic substances application and deficit irrigation in triploid watermelon. HortScience 55, 716–721. doi: 10.21273/HORTSCI14872-20

[B37] Rahmati M. Miras-Avalos J. M. Valsesia P. Lescourret F. Genard M. Davarynejad G. H.. (2018). Disentangling the effects of water stress on carbon acquisition, vegetative growth, and fruit quality of peach trees by means of the QualiTree model. Front. Plant Sci. 9. doi: 10.3389/fpls.2018.00003 PMC5788000 29416545

[B38] Skaggs T. H. Trout T. J. Rothfuss Y. (2010). Drip irrigation water distribution patterns: effects of emitter rate, pulsing, and antecedent water. Soil Sci. Soc Am. J. 74, 1886–1896. doi: 10.2136/sssaj2009.0341

[B39] Wang Y. Kou D. Muneer M. Fang G. Su D. (2020a). The effects of irrigation regimes on soil moisture dynamics, yield and quality of lucerne under subsurface drip irrigation. Appl. Ecol. Environ. Res. 18, 4179–4194. doi: 10.15666/aeer/1803_41794194

[B40] Wang H. Li J. Fan F. Han X. Liu S. Li Z.. (2018a). Evapotranspiration model and crop coefficient of greenhouse eggplant in north China. Chin. J. Eco-Agric. 26, 1819–1827. doi: 10.13930/j.cnki.cjea.180192

[B41] Wang X. Liu H. Zhang R. Li Y. (2014). Research on the estimating methods for reference crop evapotranspiration in hetao irrigation district. Agric. Res. Arid Areas 32, 95–101. doi: 10.7606/j.issn.1000-7601.2014.03.016

[B42] Wang Q. Wang K. Su L. Zhang J. Wei K. (2021). Effect of irrigation amount, nitrogen application rate and planting density on cotton leaf area index and yield. Trans. Chin. Soc Agric. Mach. 52, 300–312. doi: 10.6041/j.issn.1000-1298.2021.12.032

[B43] Wang S. Zhang H. Ba C. Wang Y. Huang C. Xue X.. (2018b). Effect of regulated deficit irrigation on growth and water use of pepper with mulched drip irrigation. Agric. Res. Arid Areas 36, 31–38. doi: 10.7606/j.issn.1000-7601.2018.03.05

[B44] Wang Y. Zhang X. Lu L. Gu N. Wang Z. Liu M.. (2020b). Estimation of crop coefficient and evapotranspiration of summer maize by path analysis combined with BP neural network. Trans. Chin. Soc Agric. Eng. 36, 109–116. doi: 10.11975/j.issn.1002-6819.2020.07.012

[B45] Wang W. Zhao X. Zhang M. Li H. Lan H. Shi Y. (2019). Public perception of water resources and water-saving intention in arid inland river basins of northwestern China: a case study of the hexi corridor in gansu province. China Popul. Resour. Environ. 29, 148–157. doi: 10.12062/cpre.20190512

[B46] Wei Y. Ma Y. Feng D. Xiong J. Zhang Y. Zhang Y. (2017). Characteristics of water dynamic response and growth of root and crown of maize under drip irrigation of regulated deficit irrigation. Trans. Chin. Soc Agric. Mach. 48, 180–188. doi: 10.6041/j.issn.1000-1298.2017.07.023

[B47] Wright J. (1982). New evapotranspiration crop coefficients. J. Irrig. Drain. Division-ASCE 108, 57–74. doi: 10.1061/jrcea4.0001372

[B48] Wu G. Wang K. (2008). Effect of water treatment on vegetative and fruit growth and fruit quality of watermelon. J. Agric. Univ. Hebei 31, 37–41. doi: 10.3969/j.issn.1000-1573.2008.02.008

[B49] Yang H. Liu H. Zheng J. Huang Q. (2018). Effects of regulated deficit irrigation on yield and water productivity of chili pepper (*Capsicum annuum* l.) in the arid environment of Northwest China. Irrig. Sci. 36, 61–74. doi: 10.1007/s00271-017-0566-4

[B50] Yang W. Zhao J. Zhao Y. Wang Q. (2022). Factors affecting evapotranspiration analyzed based on a structural equation model. J. Tsinghua Univ. (Science Technology) 62, 581–588. doi: 10.16511/j.cnki.qhdxxb.2021.22.031

[B51] Yavuz D. Seymen M. Yavuz N. Çoklar H. Ercan M. (2021). Effects of water stress applied at various phenological stages on yield, quality, and water use efficiency of melon. Agric. Water Manage. 246, 106673. doi: 10.1016/j.agwat.2020.106673

[B52] Yun W. Hou Q. Li J. Miao B. Feng X. (2015). Yield prediction of sunflower based on crop coefficient and water production function. J. Appl. Meteorol. Sci. 26, 705–713. doi: 10.11898/1001-7313.20150607

[B53] Zeng J. (2018). Water consumption characteristics and irrigation schedule of mountain apple tree of surge-root irrigation in loess hilly-gullied area of northern shaanxi (Shangxi: Xi’an University of Technology).

[B54] Zhang B. Liu G. Dai Y. Zhou J. Li Y. Chen Z.. (2009). Effect of irrigation frequency on plant growth, yield and fruit quality of greenhouse mini watermelon. China Cucurbits Veg. 91, 7–9. doi: 10.3969/j.issn.1673-2871.2009.06.003

[B55] Zhang Z. Liu S. Jia S. Du F. Qi H. Li J.. (2021). Precise soil water control using a negative pressure irrigation system to improve the water productivity of greenhouse watermelon. Agric. Water Manage. 258, 107144. doi: 10.1016/j.agwat.2021.107144

[B56] Zhang Y. Zhang L. Zhang H. Song C. Lin G. Han W. (2019). Crop coefficient estimation method of maize by UAV remote sensing and soil moisture monitoring. Trans. Chin. Soc Agric. Eng. 35, 83–89. doi: 10.11975/j.issn.1002-6819.2019.01.010

[B57] Zhao X. Lei O. Zhao P. Zhang C. (2018). Effects of size and microclimate on whole-tree water use and hydraulic regulation in schima superba trees. PeerJ 6, e5164. doi: 10.7717/peerj.5164 30002983 PMC6037140

[B58] Zheng J. (2009). Research on high efficient water use mechanism and irrigation model of mini-watermelon in greenhouse (Shangxi: Northwest A & F University).

[B59] Zhou C. Zhang H. Li F. Wang Y. Wang Y. Wang Z. (2022). Deficit mulched drip irrigation improved yield and quality while reduced water consumption of *Isatis indigotica* in a cold and arid environment. Front. Plant Sci. 13. doi: 10.3389/fpls.2022.1013131 PMC9563244 36247605

[B60] Zuniga M. Ortega-Farias S. Fuentes S. Riveros-Burgos C. Poblete-Echeverria C. (2018). Effects of three irrigation strategies on gas exchange relationships, plant water status, yield components and water productivity on grafted carménère grapevines. Front. Plant Sci. 9. doi: 10.3389/fpls.2018.00992 PMC6052738 30050549

