# Supplementary figures and images for: Crop coefficient determination and evapotranspiration estimation of watermelon under water deficit in a cold and arid environment
Source: Front Plant Sci. 2023 Jun 16;14:1153835. doi: 10.3389/fpls.2023.1153835 (PMC10312094; doi:10.3389/fpls.2023.1153835)

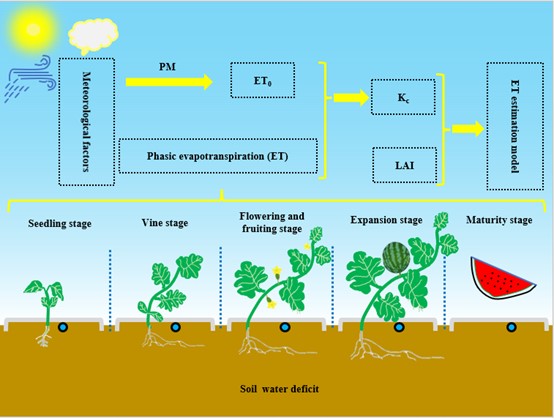

Supplement: Supplementary file 1 [file Image_1.jpeg]
